# Neurological adverse events associated with oxaliplatin: A pharmacovigilance analysis based on FDA adverse event reporting system

**DOI:** 10.3389/fphar.2024.1431579

**Published:** 2024-07-09

**Authors:** Xianglin Pan, Xiangtian Xiao, Yiling Ding, Yamin Shu, Wenting Zhang, Liu Huang

**Affiliations:** ^1^ Department of Pharmacy, The Central Hospital of Wuhan, Tongji Medical College, Huazhong University of Science and Technology, Wuhan, China; ^2^ Department of Oncology, Tongji Hospital, Tongji Medical College, Huazhong University of Science and Technology, Wuhan, China; ^3^ Department of Pharmacy, Tongji Hospital, Tongji Medical College, Huazhong University of Science and Technology, Wuhan, China

**Keywords:** oxaliplatin, neurological adverse events, the food and drug adverse event reporting system, date mining, adverse event signals

## Abstract

**Objective:**

This study aimed to explore the neurological adverse events of oxaliplatin through the Food and Drug Administration Adverse Event Reporting System (FAERS) database and to provide reference for safe clinical drug use.

**Methods:**

The adverse events report data of oxaliplatin from the first quarter of 2019 (1 January 2019) to the third quarter of 2023 (30 September 2023) were extracted from FAERS database, and the adverse events signal intensity was determined using the reporting odds ratio, proportional reporting ratio, information component, and empirical Bayes geometric mean methods. Time-to-onset and univariate logistic regression analysis were performed to describe the characteristics and risk factors of oxaliplatin-associated neurological adverse events.

**Results:**

A total of 4,471 cases of oxaliplatin-associated neurological adverse events were identified, with 318 neurological adverse events being documented, among which 87 adverse events satisfied the thresholds of four methodologies. The median time-to-onset of oxaliplatin-associated neurological adverse events was 2 days (interquartile range 0–36 days). Among the factors significantly influencing oxaliplatin-related neurological adverse events, male sex and combination medication decreased the risk of neurological adverse events, while higher cumulative dose increased the risk.

**Conclusion:**

The real-world neurotoxicity spectrum of oxaliplatin and its characteristics and influencing factors were obtained through data mining of FAERS, providing valuable insights for healthcare professionals to effectively manage the risk of neurological adverse events associated with oxaliplatin in clinical practice.

## 1 Introduction

Oxaliplatin, a platinum-based chemotherapeutic agent, is effective in the treatment of digestive system tumors, such as colon cancer, stomach cancer and liver cancer. Oxaliplatin-based therapy, including FOLFOX (oxaliplatin in combination with folinic acid and 5-fluoruracil) and CAPOX (oxaliplatin and capecitabine) are widely used in the treatment of colon cancer (Mine et al., 2022). Oxaliplatin exerts its anti-cancer effect by interfering with tumor cell proliferation through the formation of DNA-platinum adducts (Yang et al., 2021). However, oxaliplatin is also likely to interact with normal cells with high proliferation rates, thereby altering their physiological characteristics and causing adverse side effects (Oun et al., 2018). Over the years, many studies have highlighted the harmful effects of oxaliplatin on different organs and tissues, including neurotoxicity, gastrointestinal reactions, and myelosuppression (Branca et al., 2021).

Neurological adverse events (AEs) are the most prominent dose-limiting and disabling side effects of oxaliplatin and affect over 80% of treated patients (Seretny et al., 2014). The neurological AEs of oxaliplatin are mainly manifested as cold-sensitive paresthesia, dysesthesia and motor symptoms, preferentially in hands and feet resembling a stocking-and-glove pattern (Ventzel et al., 2016). Paresthesia includes numbness, prickling, tingling, or tickling (Oun et al., 2018). Dysesthesia exhibits as pain from stimulation that does not normally cause pain or other abnormal sensation of touch (Oun et al., 2018). Motor symptoms include fasciculations and prolonged muscular contractions (Yang et al., 2021).

Neurological AEs of oxaliplatin are clinically important for several reasons. First, as the specific dose-limiting toxicity of oxaliplatin, neurological AEs may lead to reduction of oxaliplatin dose or early discontinuation of therapy, which may affect chemotherapy effectiveness of patients (Marcotti et al., 2023). Second, oxaliplatin-induced neurological AEs may ultimately lead to long-term neurological deficits such as sensory loss and changes in proprioception, which may affect patients’ daily activities and persist for months or even years (Mols et al., 2013). Third, oxaliplatin-induced neurological AEs is frequent and affects over 80% of treated patients (Velasco et al., 2014). Fourth, effective treatment and prevention strategies of neurological AEs are limited. Duloxetine is the only drug moderately recommended for the treatment of oxaliplatin-induced neuropathy by the American Society of Clinical Oncology, but adverse drug reactions make it controversial. There is no agent recommended for the prevention of oxaliplatin-induced neuropathy (Loprinzi et al., 2020). Therefore, detailed investigation of oxaliplatin-associated neurological AEs is urgently needed.

Although oxaliplatin-associated neurological AEs have been described in some clinical trials, the detailed analysis of oxaliplatin-associated neurological AEs based on post-marketing surveillance data has not been reported. The Food and Drug Adverse Event Reporting System (FAERS) is a spontaneous reporting system for adverse drug events and widely used to identify pharmacovigilance risk signals for post-marketing drugs. To better understand the relationship of oxaliplatin and its neurological AEs, this study analyzed the neurological AEs of oxaliplatin from the first quarter (Q1) of 2019 (1 January 2019) to the 2023 Q3 (30 September 2023) in FAERS database. The disproportionality analysis was used for quantitative measurement of AEs signal intensity. Furthermore, univariate logistic regression and time-to-onset (TTO) analysis were performed to describe the characteristics and risk factors of oxaliplatin-associated neurological AEs.

## 2 Methods

### 2.1 Study design and data sources

The present study employed an observational, retrospective pharmacovigilance approach utilizing the publicly accessible FAERS database, which was specifically designed to facilitate post-marketing surveillance and enhance drug safety signaling (Jedlowski and DuPont, 2023). Adverse events (AEs) in the FAERS database were categorized based on drug exposures in individual patient cases and classified using standardized Medical Dictionary for Regulatory Activities (MedDRA^®^) preferred term (PT) codes (Setyawan et al., 2021). MedDRA^®^ employs a hierarchical structure that facilitates the grouping of PTs into different higher levels, including system organ class (SOC), high level group term (HLGT), and high level term (HLT). The generic name (oxaliplatin) and trade name (eloxatin) were utilized as key fields to filter cases spanning from the first quarter (Q1) of 2019 (1 January 2019) to the 2023 Q3 (30 September 2023). The cases identified with oxaliplatin as the primary suspect (PS) role code were specifically chosen, and all PTs falling under the SOC of nervous system disorders (n = 4,471) in MedDRA^®^ (version 26.1) were designated for subsequent analysis in this study. The FDA’s recommendation was followed to retain only the most recent case version in the event of multiple reports being detected (Shu et al., 2023). Repetitive reports were further eliminated based on the unique case ID and the characteristics of each individual case. The relevant data, such as gender, age, body weight, country of reporting, indication for use, cumulative dose, outcome and time-to-onset duration will be systematically collected and analyzed when available. Factors such as sex, weight, age, cumulative dose, and whether the individual had combined medication were defined as exposure factors for oxaliplatin-related neurological AEs. The univariate logistic regression analysis was conducted to determine the odds ratio (OR) for oxaliplatin-related neurological AEs across various exposures (Zhou et al., 2023). The detailed procedure was depicted in Supplementary Figure S1.

### 2.2 Signal mining

In case-control studies, cases were defined as reports that exhibited the AE of interest, while controls comprised all other AE reports except for the one of interest. Subsequently, cases and controls were stratified based on their exposure or non-exposure to the drug under investigation. Disproportionality analyses, based on the principles of calculations using a two by two table, were widely employed to identify drug-associated AEs (signals) that exhibited higher reporting frequencies than expected (Caster et al., 2020). This was achieved by estimating the proportion of specific AEs occurring between a particular drug and all other drugs, utilizing statistical measures such as reporting odds ratio (ROR), proportional reporting ratio (PRR), information component (IC), and empirical Bayes geometric mean (EBGM). We conducted disproportionality analyses at both PT and SOC levels to investigate the correlation of oxaliplatin across different hierarchical levels. The associations between drugs and AEs were assessed using four calculation methods (Supplementary Table S1). In order to mitigate the risk of false positives, consideration was given to AE overreporting when all four algorithmic criteria were simultaneously met.

### 2.3 Time-to-onset

The median, quartiles, and Weibull shape parameter (WSP) were employed to assess the time-to-onset (TTO) data for oxaliplatin-related neurological AEs (Cornelius et al., 2012). TTO was calculated from the initiation of a subject’s initial prescription until the occurrence of the AEs using data from the FAERS database. To ensure the precision of our calculation, we excluded cases lacking complete year, month, and day data as well as those with an event date preceding the drug start date. The WSP test was utilized for statistical analysis of TTO data and could elucidate the non-constant incidence rate of AEs (i.e., the dynamic risk of increase or decrease over time) (Sauzet et al., 2013). We calculated the median TTO and WSP of AEs, which occurred in at least 100 reported cases after the initiation of oxaliplatin therapy, aiming to prognosticate the risk associated with these AEs over time.

## 3 Results

### 3.1 Descriptive analysis

**FIGURE 1:**
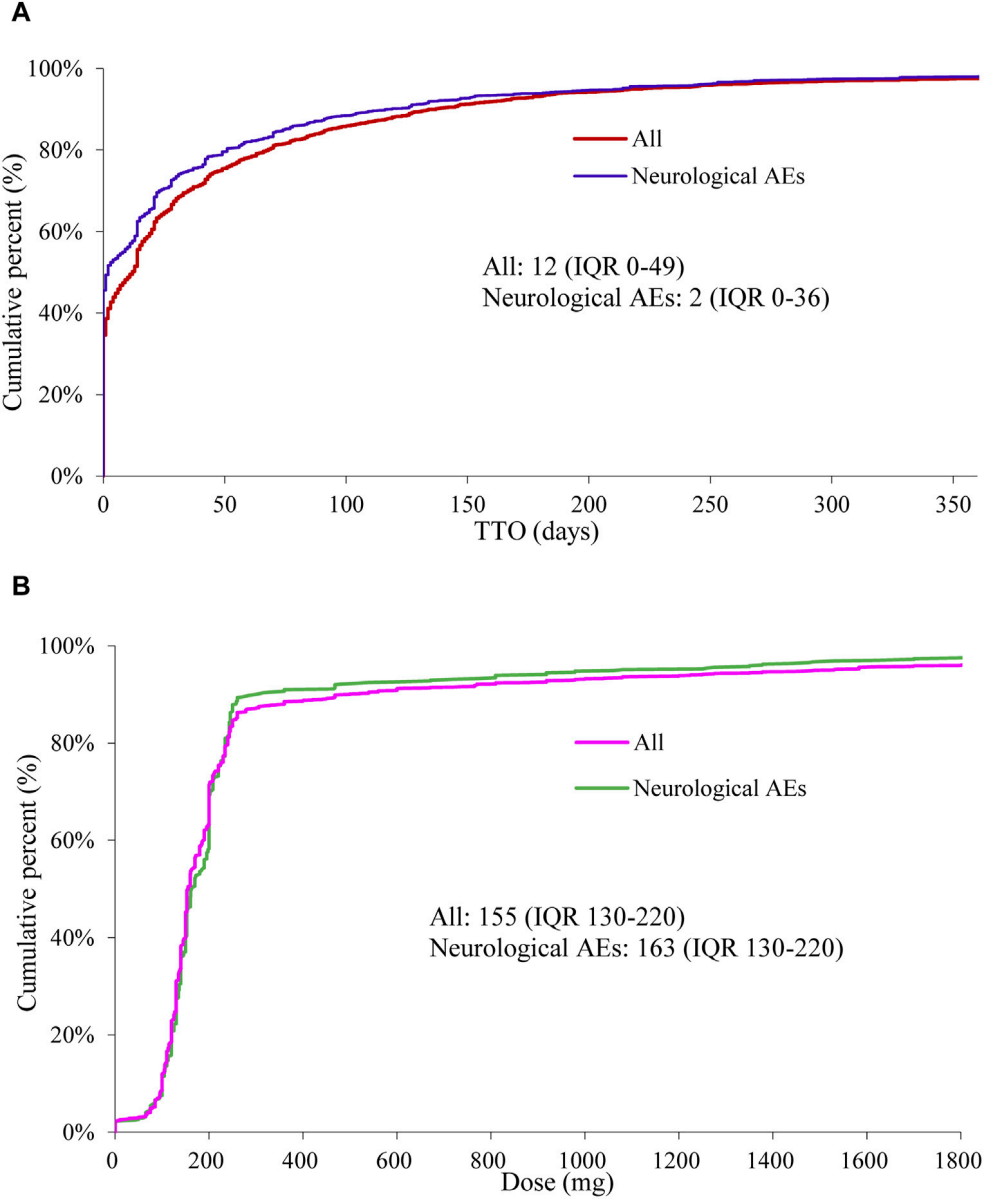
Fitting curves of time-to-onset (TTO) and cumulative percent of oxaliplatin-associated AEs (neurological AEs or overall AEs) **(A)**. Fitting Curves of dose and cumulative percent of oxaliplatin-associated AEs (neurological AEs or overall AEs) **(B)**.

A total of 14,077 reports related to oxaliplatin were documented in the FAERS database during the study period, out of which 4,471 reports specifically reported neurological AEs associated with oxaliplatin. We have summarized the clinical characteristics of these reports, and a detailed description could be found in Table 1. In reports of oxaliplatin-related neurological AEs, the number of male cases 2094 (51.81%) exceeded that of females 1948 (48.19%). The median age at onset was 64 years (interquartile range [IQR] 56–71), and the median weight was 69 kg (IQR 57–78). France accounted for the highest number of reported cases with 740 (16.67%), while colorectal cancer remained the primary indication for its usage, comprising 2,312 cases (59.28%). The study documented serious cases of neurological and overall AEs, including fatalities in 5.25% (222 out of 4,226 patients) and 8.75% (1,149 out of 13,136 patients), respectively. We drew a neurotoxicity radiographic column chart depicting experiences of death events (Supplementary Figure S2). The proportions of cerebellar infarction and metastases to meninges were 60.00% (3/5) and 44.44% (4/9), respectively, resulting in unfortunate patient mortality. However, it should be noted that the observed deaths were likely attributable to disease progression. The median TTO for neurological AEs was 2 days (IQR 0–36), which is shorter compared to the overall AEs occurring at a median of 12 days (IQR 0–49) (Figure 1A). In contrast, the median cumulative dose of neurological AEs was 163 mg (IQR 130–220), slightly exceeding the overall AEs observed at a dosage of 155 mg (IQR 130–220) (Figure 1B). The reports of oxaliplatin-related neurological AEs were submitted by healthcare professionals and non-health professionals in 4,308 (96.90%) and 138 (3.10%) cases, respectively.

**TABLE 1:** Clinical characteristics of patients with oxaliplatin-associated nervous system disorders.

Characteristics	Oxaliplatin induced neurological AEs (n = 4,471)		Oxaliplatin induced overall AEs (n = 14,077)	
	Available number	Value	Available number	Value
Sex, n (%)	4,042 (90.40%)	-	12,436 (88.34%)	-
Female	-	1948 (48.19%)	-	5,577 (44.85%)
Male	-	2094 (51.81%)	-	6,859 (55.15%)
Age (years), n (%)	3,915 (87.56%)	-	12,006 (85.29%)	-
≤65	-	2,184 (55.79%)	-	6,725 (56.01%)
>65	-	1731 (44.21%)	-	5,281 (43.99%)
Median (IQR)	-	64 (56–71)	-	64 (55–71)
Weight (Kg), n (%)	1,694 (37.89%)	-	5,226 (37.12%)	-
≤70	-	937 (55.31%)	-	2,863 (54.78%)
>70	-	757 (44.69%)	-	2,363 (45.22%)
Median (IQR)	-	69 (57–78)	-	69 (58–80)
Reported countries, n (%)	4,440 (99.31%)	-	13,993 (99.40%)	-
France	-	740 (16.67%)	-	2,520 (18.01%)
Italy	-	728 (16.40%)	-	1850 (13.22%)
United States	-	428 (9.64%)	-	1790 (12.79%)
Netherlands	-	381 (8.58%)	-	1,195 (8.54%)
Japan	-	310 (6.98%)	-	1,032 (7.38%)
Indications, n (%)	3,900 (87.23%)	-	12,343 (87.68%)	-
Colorectal cancer	-	2,312 (59.28%)	-	6,757 (54.74%)
Others	-	1,588 (40.72%)	-	5,586 (45.26%)
Outcomes, n (%)	4,471 (100%)	-	14,077 (100%)	-
Non-serious Outcome	-	245 (5.48%)	-	941 (6.68%)
Serious Outcome	-	4,226 (94.52%)	-	13,136 (93.32%)
Death	-	222 (5.25%)	-	1,149 (8.75%)
Life-threatening	-	455 (10.77%)	-	1,466 (11.16%)
Hospitalization	-	1,422 (33.65%)	-	5,101 (38.83%)
Disability	-	105 (2.48%)	-	168 (1.28%)
Other serious outcomes	-	3,166 (74.92%)	-	9,153 (69.68%)
Cumulative dose (mg)	609 (13.62%)	-	1,530 (10.87%)	-
<200	-	354 (58.13%)	-	966 (63.14%)
≥200	-	255 (41.87%)	-	564 (36.86%)
Median (IQR)	-	163 (130–220)	-	155 (130–220)
Time-to-onset (days)	2,205 (49.32%)	-	6,977 (49.56%)	-
≤30	-	1,622 (73.56%)	-	4,754 (68.14%)
>30	-	583 (26.44%)	-	2,223 (31.86%)
Median (IQR)	-	2 (0–36)	-	12 (0–49)
Reporters, n (%)	4,446 (99.44%)	-	13,976 (99.28%)	-
Health professional	-	4,308 (96.90%)	-	13,573 (97.12%)
Non-health professional	-	138 (3.10%)	-	403 (2.88%)
Reporting year, n (%)	4,471 (100%)	-	14,077 (100%)	-
2023 Q3^a^	-	656 (14.67%)	-	2,358 (16.75%)
2022	-	810 (18.12%)	-	2,682 (19.05%)
2021	-	853 (19.08%)	-	2,779 (19.74%)
2020	-	948 (21.20%)	-	2,871 (20.39%)
2019	-	1,204 (26.93%)	-	3,387 (24.06%)

^a^ The third quarter of 2023.

AEs, Adverse events; n, number of cases.

### 3.2 Exploring oxaliplatin-related neurological AEs

Throughout the duration of the study, a total of 318 distinct oxaliplatin-associated AEs pertaining to nervous system disorders were documented in the FAERS database (Supplementary Table S2). The following categories of neurological AEs had the highest number of cases: neuropathy peripheral (n = 835), paraesthesia (n = 535), neurotoxicity (n = 432), dizziness (n = 250), dysarthria (n = 207), tremor (n = 198), polyneuropathy (n = 178), loss of consciousness (n = 168), dysphonia (n = 155), hypoaesthesia (n = 151), palmar-plantar erythrodysaesthesia syndrome (n = 125), speech disorder (n = 111), and headache (n = 100). Subsequently, we utilized the entire FAERS dataset as the reference group and identified 87 PTs that satisfied the thresholds of all four methodologies (Table 2). Furthermore, we discovered an additional 137 PTs that only met the ROR threshold (Supplementary Table S3). We presented the association between the nine PTs with more than 100 cases that satisfied four methodological criteria, along with their relationships to other hierarchies in MedDRA 26.1 (Supplementary Figure S3). Additionally, neuropathy peripheral, paraesthesia, neurotoxicity, dysarthria, polyneuropathy, loss of consciousness and speech disorder were classified as primary SOC within nervous system disorders. At the level of SOC, nervous system disorders (4,471, ROR 1.90, PRR 1.97, IC 0.69, EBGM 1.61) met the thresholds for ROR and IC criteria, while blood and lymphatic system disorders (2,594, ROR 4.08, PRR 4.26, IC 1.80, EBGM 3.49) and hepatobiliary disorders (1,321, ROR 3.63, PRR 3.85, IC 1.75, EBGM 3.37) reached the thresholds for all four methodological approaches (Supplementary Table S4).

**TABLE 2:** Signal strength of reports of oxaliplatin-related neurological AEs at the Preferred Term (PT) level in FAERS database.

PTs	Cases	ROR (95% two-sided CI)	PRR (χ^2^)	IC (IC025)	EBGM (EBGM05)
Neuropathy peripheral	835	13.30 (12.4–14.28)	12.57 (8,723.86)	3.60 (3.50)	12.30 (11.46)
Paraesthesia	535	5.98 (5.48–6.52)	5.79 (2,109.27)	2.50 (2.38)	5.73 (5.26)
Neurotoxicity	432	33.58 (30.43–37.07)	32.58 (12,442.20)	4.84 (4.70)	30.68 (27.80)
Dysarthria	207	10.63 (9.25–12.21)	10.49 (1742.94)	3.29 (3.09)	10.29 (8.96)
Polyneuropathy	178	20.66 (17.77–24.02)	20.41 (3,161.81)	4.15 (3.92)	19.67 (16.91)
Loss of consciousness	168	2.42 (2.08–2.82)	2.40 (137.78)	1.24 (1.02)	2.40 (2.06)
Dysphonia	155	4.18 (3.57–4.90)	4.15 (367.90)	2.00 (1.77)	4.12 (3.51)
Palmar-plantar erythrodysaesthesia syndrome	125	7.83 (6.55–9.35)	7.77 (726.68)	2.85 (2.59)	7.66 (6.42)
Speech disorder	111	3.67 (3.04–4.42)	3.65 (212.18)	1.81 (1.54)	3.63 (3.01)
Paraesthesia oral	86	10.21 (8.24–12.64)	10.15 (696.00)	3.16 (2.84)	9.97 (8.05)
Muscle rigidity	72	10.03 (7.94–12.67)	9.99 (571.28)	3.11 (2.77)	9.81 (7.77)
Peripheral sensory neuropathy	65	18.53 (14.46–23.75)	18.45 (1,035.64)	3.81 (3.44)	17.84 (13.92)
Dysaesthesia pharynx	61	2081.15 (1,182.7–3,662.11)	2072.14 (24,923.62)	5.73 (5.23)	409.78 (232.87)
Encephalopathy	52	3.34 (2.54–4.39)	3.33 (84.41)	1.64 (1.24)	3.32 (2.52)
Presyncope	51	3.59 (2.73–4.73)	3.58 (94.35)	1.74 (1.33)	3.56 (2.70)
Altered state of consciousness	38	2.85 (2.07–3.93)	2.85 (45.38)	1.40 (0.93)	2.84 (2.06)
Dysaesthesia	38	22.26 (16.08–30.82)	22.20 (737.45)	3.77 (3.29)	21.32 (15.40)
Hepatic encephalopathy	38	7.25 (5.26–9.99)	7.23 (201.32)	2.59 (2.12)	7.15 (5.19)
Hypoaesthesia oral	36	4.38 (3.15–6.09)	4.37 (92.91)	1.95 (1.47)	4.34 (3.13)
Paralysis	33	3.99 (2.83–5.62)	3.98 (73.07)	1.82 (1.32)	3.96 (2.81)
Band sensation	32	34.33 (23.99–49.12)	34.25 (968.08)	4.00 (3.48)	32.16 (22.48)
Posterior reversible encephalopathy syndrome	31	4.79 (3.36–6.83)	4.78 (91.95)	2.04 (1.52)	4.75 (3.33)
Trismus	31	11.35 (7.95–16.20)	11.32 (285.47)	3.03 (2.51)	11.10 (7.77)
Hyperammonaemic encephalopathy	28	25.34 (17.33–37.06)	25.30 (622.55)	3.70 (3.14)	24.15 (16.52)
Guillain-Barre syndrome	26	8.71 (5.91–12.83)	8.69 (174.08)	2.69 (2.12)	8.56 (5.81)
Pharyngeal paraesthesia	26	16.78 (11.35–24.81)	16.75 (372.80)	3.32 (2.75)	16.25 (10.99)
Vocal cord paralysis	26	26.60 (17.92–39.46)	26.55 (607.65)	3.68 (3.10)	25.28 (17.04)
Papilloedema	23	9.69 (6.41–14.65)	9.68 (175.65)	2.75 (2.14)	9.52 (6.30)
Motor dysfunction	21	3.47 (2.26–5.34)	3.47 (36.68)	1.57 (0.94)	3.45 (2.25)
Amaurosis fugax	20	40.50 (25.68–63.87)	40.44 (712.75)	3.71 (3.04)	37.54 (23.80)
Muscle contractions involuntary	18	9.98 (6.26–15.92)	9.97 (142.48)	2.67 (1.98)	9.80 (6.14)
Tonic clonic movements	18	23.73 (14.79–38.08)	23.70 (373.98)	3.33 (2.63)	22.69 (14.14)
Monoparesis	16	15.52 (9.43–25.52)	15.50 (210.63)	2.96 (2.22)	15.07 (9.16)
Hyporeflexia	15	18.13 (10.83–30.35)	18.11 (234.19)	3.01 (2.26)	17.52 (10.47)
Toxic leukoencephalopathy	15	21.99 (13.11–36.87)	21.96 (287.74)	3.13 (2.37)	21.10 (12.58)
Immune effector cell-associated neurotoxicity syndrome	14	3.58 (2.11–6.05)	3.57 (25.78)	1.50 (0.73)	3.56 (2.10)
Leukoencephalopathy	14	7.06 (4.16–11.96)	7.05 (71.69)	2.22 (1.44)	6.97 (4.11)
Nystagmus	14	5.08 (3.00–8.61)	5.08 (45.44)	1.89 (1.11)	5.04 (2.98)
Petit mal epilepsy	14	5.36 (3.17–9.08)	5.36 (49.14)	1.95 (1.17)	5.31 (3.14)
Acute polyneuropathy	13	79.88 (44.51–143.37)	79.81 (874.68)	3.45 (2.60)	69.14 (38.52)
Hypertonia	13	6.24 (3.61–10.79)	6.24 (56.49)	2.07 (1.26)	6.17 (3.57)
Peripheral sensorimotor neuropathy	13	18.78 (10.80–32.68)	18.76 (210.88)	2.92 (2.11)	18.13 (10.42)
Toxic encephalopathy	12	3.61 (2.05–6.37)	3.61 (22.48)	1.47 (0.63)	3.59 (2.03)
Paraparesis	11	11.11 (6.11–20.19)	11.10 (98.93)	2.45 (1.57)	10.88 (5.99)
Tunnel vision	11	9.89 (5.45–17.97)	9.89 (86.19)	2.37 (1.49)	9.72 (5.35)
Neuromyotonia	10	196.12 (94.56–406.76)	195.98 (1,400.99)	3.22 (2.19)	141.82 (68.38)
Peripheral nerve injury	10	18.47 (9.83–34.73)	18.46 (159.39)	2.68 (1.75)	17.85 (9.50)
Toxic neuropathy	10	32.07 (16.92–60.77)	32.05 (282.99)	2.91 (1.97)	30.21 (15.94)
Anal sphincter atony	9	52.74 (26.55–104.79)	52.71 (413.77)	2.92 (1.92)	47.86 (24.09)
Cholinergic syndrome	9	22.17 (11.37–43.22)	22.15 (174.22)	2.66 (1.68)	21.27 (10.91)
Cold dysaesthesia	9	4,588.81 (581.31–36223.5)	4,585.87 (4,125.49)	3.14 (1.82)	459.49 (58.21)
Hemiparaesthesia	9	20.39 (10.47–39.71)	20.38 (159.50)	2.63 (1.65)	19.64 (10.08)
Infusion site hypoaesthesia	9	111.92 (54.39–230.32)	111.85 (810.77)	3.04 (1.99)	91.90 (44.66)
Injection site paraesthesia	9	9.58 (4.95–18.53)	9.57 (67.83)	2.20 (1.23)	9.42 (4.87)
Metastases to meninges	9	5.63 (2.92–10.86)	5.63 (33.87)	1.78 (0.82)	5.58 (2.89)
Quadriplegia	9	8.59 (4.45–16.61)	8.59 (59.34)	2.12 (1.15)	8.46 (4.38)
Atypical haemolytic uraemic syndrome	8	9.23 (4.58–18.57)	9.22 (57.61)	2.09 (1.06)	9.08 (4.51)
Foaming at mouth	8	8.68 (4.31–17.46)	8.67 (53.40)	2.05 (1.02)	8.54 (4.25)
Focal dyscognitive seizures	8	6.05 (3.01–12.15)	6.05 (33.31)	1.78 (0.75)	5.99 (2.98)
Neuromyopathy	8	12.36 (6.13–24.93)	12.35 (81.50)	2.27 (1.23)	12.08 (5.99)
Oromandibular dystonia	8	18.88 (9.32–38.25)	18.87 (130.57)	2.48 (1.44)	18.23 (9.00)
Peripheral motor neuropathy	8	9.25 (4.60–18.61)	9.24 (57.77)	2.09 (1.06)	9.10 (4.52)
Toxic optic neuropathy	8	23.04 (11.34–46.81)	23.03 (161.30)	2.55 (1.51)	22.08 (10.87)
Ophthalmoplegia	7	5.88 (2.79–12.39)	5.88 (28.00)	1.67 (0.57)	5.82 (2.76)
Optic ischaemic neuropathy	7	6.24 (2.96–13.15)	6.24 (30.40)	1.71 (0.62)	6.17 (2.93)
Uraemic encephalopathy	7	34.99 (16.26–75.26)	34.97 (216.15)	2.53 (1.40)	32.79 (15.24)
Apraxia	6	6.55 (2.93–14.65)	6.55 (27.84)	1.64 (0.45)	6.48 (2.89)
Cranial nerve disorder	6	15.29 (6.79–34.45)	15.29 (77.78)	2.10 (0.90)	14.87 (6.60)
Demyelinating polyneuropathy	6	10.69 (4.76–24.01)	10.69 (51.62)	1.93 (0.74)	10.49 (4.67)
Hyporesponsive to stimuli	6	7.03 (3.14–15.74)	7.03 (30.60)	1.69 (0.50)	6.95 (3.10)
Oral dysaesthesia	6	48.55 (21.01–112.18)	48.53 (255.01)	2.40 (1.17)	44.39 (19.21)
Vestibular neuronitis	6	18.65 (8.26–42.13)	18.64 (96.64)	2.17 (0.97)	18.02 (7.98)
Cerebellar infarction	5	6.85 (2.83–16.56)	6.85 (24.64)	1.52 (0.23)	6.77 (2.80)
Decreased eye contact	5	22.76 (9.29–55.75)	22.75 (99.52)	2.02 (0.71)	21.82 (8.91)
Decreased vibratory sense	5	19.45 (7.96–47.53)	19.45 (84.28)	1.98 (0.67)	18.77 (7.68)
Diaphragmatic paralysis	5	15.45 (6.34–37.61)	15.44 (65.54)	1.91 (0.60)	15.02 (6.17)
Hereditary motor and sensory neuropathy	5	57.92 (22.96–146.10)	57.90 (251.07)	2.19 (0.83)	52.10 (20.65)
Lhermitte’s sign	5	24.04 (9.80–58.97)	24.03 (105.41)	2.04 (0.72)	23 (9.38)
Osmotic demyelination syndrome	5	10.75 (4.43–26.08)	10.75 (43.30)	1.76 (0.46)	10.55 (4.35)
Resting tremor	5	10.15 (4.19–24.61)	10.15 (40.44)	1.74 (0.43)	9.97 (4.11)
Transient aphasia	5	36.41 (14.69–90.22)	36.40 (160.64)	2.12 (0.79)	34.04 (13.74)
Axonal and demyelinating polyneuropathy	4	46.33 (16.65–128.97)	46.32 (162.59)	1.87 (0.37)	42.55 (15.28)
Cerebral vasoconstriction	4	9.27 (3.45–24.91)	9.26 (28.96)	1.48 (0.02)	9.12 (3.39)
Neurosarcoidosis	4	11.65 (4.32–31.39)	11.65 (38.06)	1.57 (0.11)	11.41 (4.23)
Pharyngeal dyskinesia	4	145.62 (47.93–442.47)	145.58 (446.73)	1.95 (0.36)	113.45 (37.34)
Pseudostroke	4	20.59 (7.58–55.96)	20.59 (71.65)	1.73 (0.26)	19.83 (7.30)
Tongue paralysis	4	17.13 (6.32–46.41)	17.13 (58.77)	1.69 (0.22)	16.60 (6.13)

ROR, reporting odds ratio; CI, confidence interval; PRR, proportional reporting ratio; χ^2^.

chi-squared; IC, information component; IC025, the lower limit of 95% CI, of the IC; EBGM, empirical Bayesian geometric mean; EBGM05, the lower limit of 95% CI, of EBGM.

**FIGURE 2:**
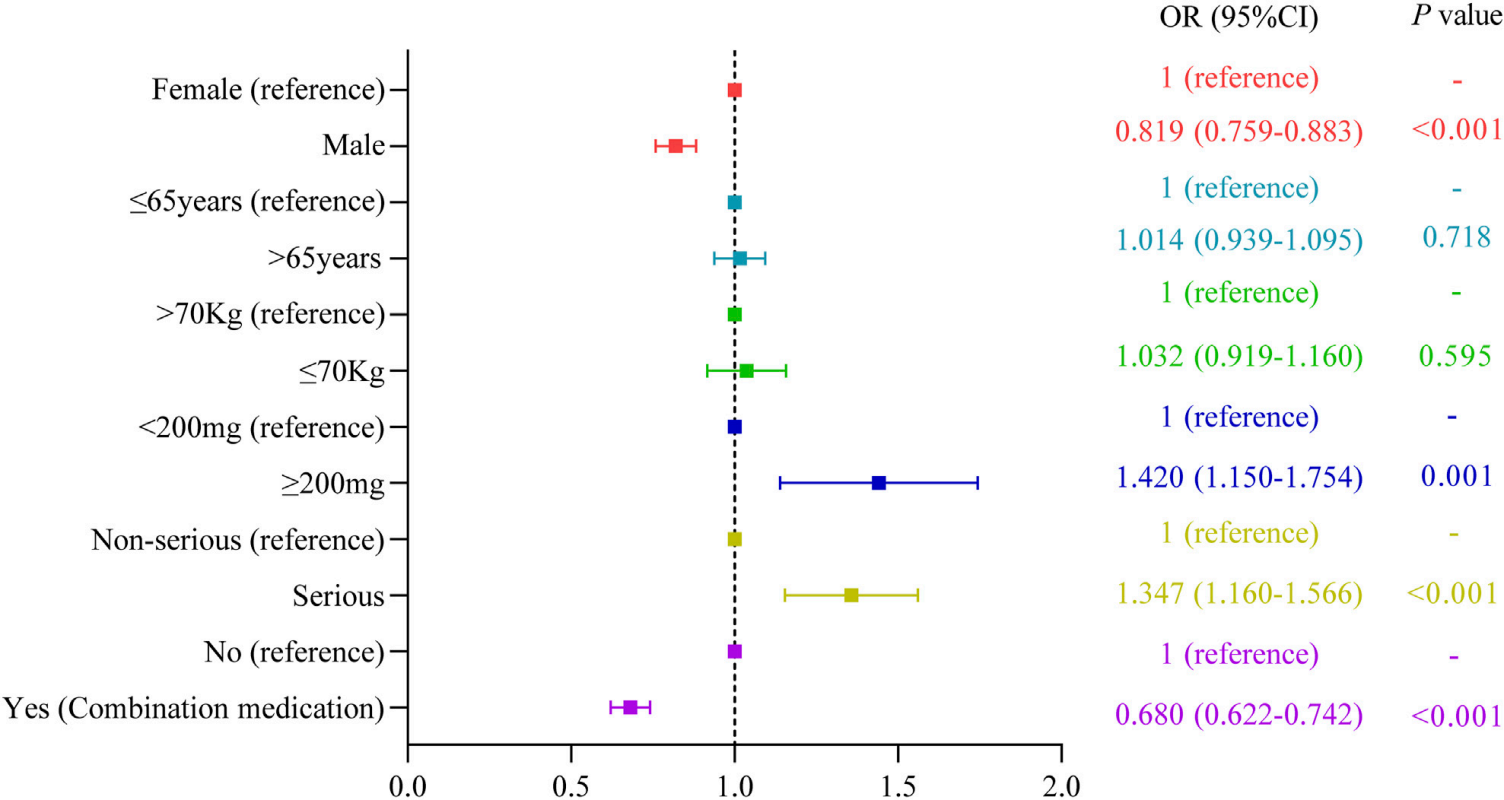
Univariate logistic regression analysis of oxaliplatin-associated neurological AEs.

**FIGURE 3:**
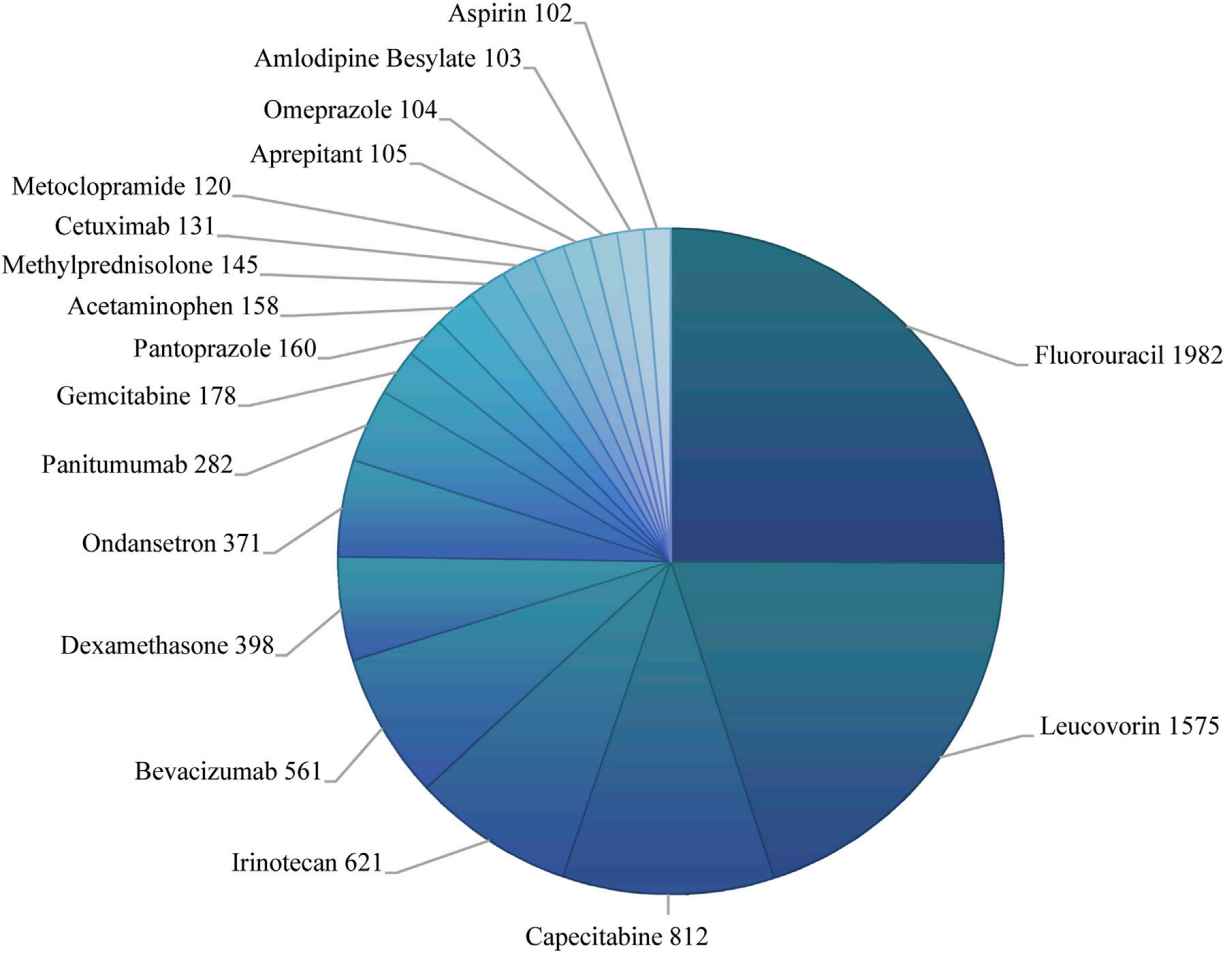
Co-administered drugs of oxaliplatin-associated neurological AEs.

We further investigated potential factors influencing the occurrence of oxaliplatin-related neurological AEs by conducting univariate logistic regression analysis using the complete dataset of oxaliplatin reports (Figure 2). The factors significantly influencing oxaliplatin-related neurological AEs were sex (male: OR = 0.819 [0.759–0.883], *p* < 0.001), cumulative dose ≥200 mg (OR = 1.420 [1.150–1.754], *p* = 0.001), serious outcome (OR = 1.347 [1.160–1.566], *p* < 0.001), and combination medication (OR = 0.680 [0.622–0.742], *p* < 0.001). The occurrence of oxaliplatin-related neurological AEs was not significantly influenced by other factors, as evidenced by ORs approximating 1 (*p* > 0.05). Additionally, fluorouracil, leucovorin, capecitabine, irinotecan, and bevacizumab were the five most frequently co-administered drugs of oxaliplatin-related neurological AEs, with 1982, 1,575, 812, 621, and 561 cases reported respectively (Figure 3).

### 3.3 Time-to-onset analysis

The findings from the TTO and WSP analyses conducted on 13 major PTs (≥100 cases) listed in Supplementary Table S2 have been summarized in Table 3. The median TTO of neuropathy peripheral, neurotoxicity, polyneuropathy and palmar-plantar erythrodysaesthesia syndrome associated with oxaliplatin was 28 days (IQR 0–87), 20 days (IQR 1–78), 28 days (IQR 0–66) and 49 days (IQR 6–126), respectively. However, the median TTO for other PTs was 0 days. The WSP test results for those PTs indicated that both the shape parameter β and its 95% CI upper limit were <1, suggesting an early failure pattern with a decreasing hazard of AEs over time.

**TABLE 3:** Results of time-to-onset analysis for PTs.

PTs	TTO (days)			Weibull distribution				Failure type
	Cases	-		Scale parameter		Shape parameter		
	n	Median (IQR)	Min-max	α	95% CI	β	95% CI	
Neuropathy peripheral	195	28 (0–87)	0–990	14.97	7.73–29.01	0.22	0.19–0.25	Early failure
Paraesthesia	357	0 (0–22)	0–892	0.52	0.26–1.05	0.15	0.14–0.17	Early failure
Neurotoxicity	129	20 (1–78)	0–669	13.77	7.00–27.10	0.26	0.23–0.31	Early failure
Dizziness	105	0 (0–11)	0–282	0.04	0.01–0.19	0.13	0.12–0.15	Early failure
Dysarthria	94	0 (0–21)	0–58	0.14	0.03–0.59	0.15	0.13–0.17	Early failure
Tremor	155	0 (0–21)	0–391	0.12	0.04–0.40	0.14	0.13–0.16	Early failure
Polyneuropathy	53	28 (0–66)	0–566	13.44	3.98–45.38	0.23	0.18–0.29	Early failure
Loss of consciousness	134	0 (0–6)	0–461	0.19	0.05–0.71	0.14	0.12–0.16	Early failure
Dysphonia	84	0 (0–21)	0–94	0.41	0.10–1.73	0.16	0.13–0.19	Early failure
Hypoaesthesia	75	0 (0–0)	0–768	0.01	0.00–0.07	0.13	0.11–0.16	Early failure
Palmar-plantar erythrodysaesthesia syndrome	37	49 (6–126)	0–666	50.48	24.05–105.95	0.45	0.34–0.59	Early failure
Speech disorder	82	0 (0–0)	0–251	0.00	0.00–0.02	0.14	0.12–0.16	Early failure
Headache	47	0 (0–0)	0–489	0.00	0.00–0.04	0.14	0.12–0.17	Early failure

n number of cases with available time-to-onset; IQR, interquartile range; TTO, time-to-onset.

When TTO, is 0 days, the adverse event occurred within the same day with the therapy.

## 4 Discussion

This study provided a comprehensive and systematic analysis of oxaliplatin-related neurological AEs based on real-world population from FAERS database. Consistent with previous clinical trials and literature reviews (Argyriou et al., 2006; Luo et al., 2022a; Luo et al., 2022b), the present study demonstrated a strong association of oxaliplatin exposure and neurological disorders through four methods of disproportionality analysis. In addition, this study also analyzed the influencing factors, time-to-onset, and combination medication of oxaliplatin-related neurological AEs.

### 4.1 Oxaliplatin-related neurological AEs

In this study, all AEs of oxaliplatin (n = 14,077) were analyzed at the SOC level (Supplementary Table S4). The results showed that nervous system disorders with reports of 4,471 had the second highest number of reports. The highest number of reports was general disorders and administration site conditions, but its signal intensity was weak (ROR of 0.99, PRR of 1.03, IC of −0.01, and EBGM of 1.00). Therefore, it could be inferred that the incidence of nervous system disorders was relatively high during the application of oxaliplatin. A phase III study investigated the effect of leucovorin and fluorouracil (LV5FU2) with or without oxaliplatin on patients with advanced colorectal cancer, and found that the incidence of neurosensory toxicity was significantly higher in patients with oxaliplatin treatment (68%) compared with patients without oxaliplatin treatment (12%) (de Gramont et al., 2023). As shown in Table 1, serious outcome was reported at a higher rate for oxaliplatin-related neurological AEs compared with oxaliplatin-related overall AEs (94.52% vs. 93.32%). Moreover, the univariate logistic regression analysis (Figure 2) demonstrated that the neurological AEs exhibited a higher risk of serious outcome (OR = 1.347 [1.160–1.566], *p* < 0.001). Because of the more serious outcome, healthcare professionals should pay more attention to the neurological AEs of oxaliplatin.

In this study, a total of 4,471 reports of oxaliplatin-related neurological AEs were obtained, and 318 neurological AEs were detected (Supplementary Table S2). The neuropathy peripheral was the most frequently reported oxaliplatin-related neurological AEs (n = 835, ROR = 13.30, PRR = 12.57, IC = 3.60, EBGM = 12.30), which indicated that oxaliplatin damaged to the nervous system principally as peripheral sensory neuropathy.

By analyzing reports with death outcome, it was found that the neurological AEs with a higher proportion of death outcome included posterior reversible encephalopathy syndrome (n = 31, death reports = 9, death proportion = 29.03%, ROR = 4.79), hepatic encephalopathy (n = 38, death reports = 8, death proportion = 21.05%, ROR = 7.25), immune effector cell-associated neurotoxicity syndrome (n = 14, death reports = 5, death proportion = 35.71%, ROR = 3.58), hypertonia (n = 13, death reports = 4, death proportion = 30.77%, ROR = 6.24), metastases to meninges (n = 9, death reports = 4, death proportion = 44.44%, ROR = 5.63), and cerebellar infarction (n = 5, death reports = 3, death proportion = 60.00%, ROR = 6.85). Special attention is necessary when these severe AEs with high mortality occur. Although the proportions of deaths from neuropathy peripheral (n = 835, death reports = 35, death proportion = 4.19%, ROR = 13.3) and loss of consciousness (n = 168, death reports = 15, death proportion = 8.93%, ROR = 2.42) were relatively low, the number of death reports was high because of high incidence.

### 4.2 Acute and chronic neurological AEs

According to the median TTO, neurological AEs of oxaliplatin could be categorized into two types, acute and chronic neurological AEs, which was consistent with previous reports (Kang et al., 2021). The median TTO of acute neurological AEs was 0 days, including paraesthesia, dizziness, dysarthria, tremor, loss of consciousness, dysphonia, hypoaesthesia, speech disorder, headache (Table 3). Acute neurological AEs usually occurred within hours of infusion, peaked in severity at the third day after oxaliplatin administration, and then gradually resolved (Beijers et al., 2014; Park et al., 2015). The chronic neurological AEs included neuropathy peripheral, neurotoxicity, polyneuropathy, palmar-plantar erythrodysaesthesia syndrome. The median TTO of chronic neurological AEs was 20–49 days (Table 3). Chronic neurological AEs occurred during oxaliplatin treatment and persisted for 6–12 months or even several years after termination of oxaliplatin treatment (Kang et al., 2021). Chronic neurological AEs of oxaliplatin might be the long-term consequence of its acute toxicity. Thus, the severity of acute neuropathy appeared to predict the development of chronic neurotoxicity (Park et al., 2015). Patients with more severe acute neuropathy usually had more severe chronic neurotoxicity.

It has been reported that the mechanisms of acute and chronic neurological AEs were not identical. The mechanism of acute neurological abnormalities was the transient impairment of axonal voltage-gated Na^+^ and K^+^ channels and nerve hyperexcitability caused by oxalate metabolites of oxaliplatin (Alberti et al., 2020). However, the main mechanisms responsible for the chronic neurotoxicity were death of sensory neurons caused by DNA damage, oxidative stress-induced mitochondrial damage, and glia activation-induced neuroinflammation, which resulted from accumulation of oxaliplatin in dorsal root ganglion (Kanat et al., 2017).

### 4.3 Risk factors of oxaliplatin-related neurological AEs

Identifying the risk factors for oxaliplatin-induced neurological AEs was of great significance in the individualization of chemotherapy. The results of univariate logistic regression analysis (Figure 2) showed that gender, cumulative dose, and combination medication were associated with the occurrence of oxaliplatin-related neurological AEs, while age and body weight did not influence the occurrence of oxaliplatin-related neurological AEs.

#### 4.3.1 Males had significantly decreased risk of oxaliplatin-related neurological AEs

As shown in Figure 2, males had significantly decreased risk of oxaliplatin-related neurological AEs compared with females (*p* < 0.001). There were more male cases (2094, 51.81%) than female cases (1948, 48.19%) among reports of oxaliplatin-associated neurological AEs (Table 1), but this did not mean that males had high incidence of oxaliplatin-related neurological AEs. What were the reasons for this phenomenon? Oxaliplatin was mainly used for the treatment of colorectal cancer, and our study also showed that the main indication of oxaliplatin was colorectal cancer, accounting for 59.28% of all indications of oxaliplatin. Epidemiological study suggested that men were more likely to suffer from colorectal cancer (GBD 2019 Colorectal Cancer Collaborators, 2022). Therefore, it was possible that more males accepted oxaliplatin treatment, resulting in a correspondingly greater number of oxaliplatin-related neurological AEs reports in males. The effect of gender on neurological AEs of oxaliplatin has also been reported in previous literature. Wang et al. reported that female sex was associated with increased severity of oxaliplatin-induced peripheral neuropathy in a prospective study of patients receiving standard oxaliplatin-based chemotherapy for colorectal cancer (Wang et al., 2016). A similar result was obtained in another study. Increased risk of neurological AEs was found among women receiving oxaliplatin-based chemotherapy for colon cancer (Wiela-Hojeńska et al., 2015). This study found an increased risk of oxaliplatin-related neurological AEs in females, suggesting that female patients using oxaliplatin need to pay special attention to neurological AEs.

#### 4.3.2 Higher cumulative doses were associated with higher risk of oxaliplatin-related neurological AEs

Compared with low cumulative dose (cumulative dose <200 mg), oxaliplatin with a higher cumulative dose (cumulative dose ≥200 mg) was more likely to cause neurological AEs (Figure 2). This finding supported the reported view that higher cumulative and single oxaliplatin doses were associated with higher incidence and severity of neuropathic symptoms (Velasco et al., 2014; Oun et al., 2018). Correspondingly, the number of treatment cycles was associated with oxaliplatin-induced neuropathy (Pulvers and Marx, 2017). Previous reports have shown that acute sensory symptoms and chronic sensory neuropathy caused by oxaliplatin usually occurred after cumulative doses of 550 mg/m^2^, which was equivalent to six to seven cycles (3 months) at a dose of 85 mg/m^2^ every 2 weeks (Park et al., 2013). In a clinical trial of 2,450 patients with stage III colon cancer recruited from the United States and Canada, patients accepted 12 cycles (6 months) of adjuvant oxaliplatin, fluorouracil, and leucovorin, relative to 6 cycles (3 months), were more likely to experience higher-grade neuropathy and longer times to resolution (Lee et al., 2023). Moreover, similar results have also been obtained in another randomized phase III trial conducted in Japan. This trial found that shortening adjuvant therapy (oxaliplatin in combination with capecitabine or oxaliplatin in combination with folinic acid and fluorouracil) duration from 6 to 3 months did not compromise efficacy and reduced the rate of long-lasting peripheral sensory neuropathy in patients with stage III colon cancer (Yoshino et al., 2022). Therefore, 3 months of oxaliplatin therapy might be a more appropriate treatment option than 6 months for patients with colon cancer.

#### 4.3.3 Combination medication decreased risk of oxaliplatin-related neurological AEs

It was noteworthy that cases with combination medication had significantly decreased risk of oxaliplatin-related neurological AEs compared with cases without combination medication (Figure 2). This result implied the possibility that drugs in the combination medication might mitigate oxaliplatin-related neurological AEs. There were 18 co-administered drugs with at least 102 cases (Figure 3). These co-administered drugs could be classified into three groups, including other antitumor drugs (fluorouracil, capecitabine, irinotecan, bevacizumab, panitumumab, gemcitabine, cetuximab), adjuvant drugs of chemotherapy (leucovorin, dexamethasone, ondansetron, pantoprazole, methylprednisolone, metoclopramide, aprepitant, omeprazole), and other drugs (acetaminophen, amlodipine besylate, aspirin).

There were two possible reasons why the combination drugs reduced the risk of oxaliplatin-related neurological AEs. First, combination with other antitumor drugs might increase the antitumor effect and decrease the dose of oxaliplatin, thereby reducing its neurotoxicity. Second, combination with adjuvant drugs of chemotherapy could alleviate other side effects of oxaliplatin, which improved the physical condition of patients and ultimately reduced the risk of neurological AEs. Interestingly, proton pump inhibitors (PPIs) and nonsteroidal anti-inflammatory drugs (NSAIDs) in combination medication have previously been reported to mitigate oxaliplatin-related neurological AEs. Keisuke Mine et al. found that reporting rate of peripheral neuropathy in oxaliplatin-treated patients was significantly lower when PPIs (omeprazole, pantoprazole, and rabeprazole) were used concomitantly (Mine et al., 2022).The results of their study also showed that omeprazole ameliorated oxaliplatin-induced mechanical hypersensitivity in a rat model. Another study reported that co-administration of NSAIDs was associated with a decreased risk of oxaliplatin-induced peripheral neuropathy (Kanbayashi et al., 2010; Pulvers and Marx, 2017).

Diabetes mellitus, pre-existing neuropathy, excessive alcohol intake, and renal impairment have also been reported to increase the risk of oxaliplatin-related neurological AEs (Brozou et al., 2017). Pharmacogenomics studies suggested that patients with polymorphisms in the Glutathione S-transferases genes (GSTM) were more likely to develop severe neuropathy during oxaliplatin treatment due to decreased drug detoxification (Cavaletti et al., 2011).

### 4.4 Limitations of this study

This study was based on the database of spontaneous adverse reaction reporting system. Due to the inherent nature of FAERS database, this study inevitably had several limitations. First, underreporting, misreporting, and incomplete reporting in FAERS database were difficult to avoid and might result in unquantifiable biases. Second, the incidence of adverse events cannot be calculated because the FAERS database only included patients who experienced adverse events and the total number of patients who received oxaliplatin was not available. Despite these limitations, this study had unique strengths. As a large-sample, real-world study based on the FAERS database, this study could overcome the shortcomings of relatively small sample size, limited follow-up duration, and strict selection criteria in clinical trials, and could reflect the occurrence of adverse events in actual clinical use of oxaliplatin.

## 5 Conclusion

In summary, this study comprehensively and systematically analyzed oxaliplatin-associated neurological AEs through the FAERS database. The following important information was obtained from this study. 1) The neurotoxicity spectrum of oxaliplatin and its characteristics were illustrated, and neuropathy peripheral was the most frequently reported oxaliplatin-associated neurological AEs. 2) The results of univariate logistic regression analysis showed that combination medication decreased the risk of neurological AEs, while female sex and higher cumulative dose increased risk of neurological AEs. 3) The TTO of 13 major PTs for oxaliplatin neurological AEs was estimated, and WSP test showed that these PTs exhibited an early failure pattern with a decreasing hazard of AEs over time. This study provided valuable evidence for healthcare professionals to recognize and mitigate the oxaliplatin-associated neurological AEs, which will contribute to the safe and rational use of oxaliplatin in clinical practice.

## Data Availability

The original contributions presented in the study are included in the article/Supplementary Material, further inquiries can be directed to the corresponding authors.

## Supplementary material

SUPPLEMENTARY TABLE S1 Four algorithms used to assess potential associations between drug and AEs.

SUPPLEMENTARY TABLE S2 All neurological AEs in oxaliplatin users from FAERS database.

SUPPLEMENTARY TABLE S3 ROR information of neurological AEs in oxaliplatin users from FAERS database.

SUPPLEMENTARY TABLE S4 Signal strength of reports of oxaliplatin at the system organ class (SOC) level in FAERS database.

SUPPLEMENTARY FIGURE S1 The process of data extraction, processing, and analysis from the Food and Drug Administration Adverse Event Reporting System (FAERS) database.

SUPPLEMENTARY FIGURE S2 Radiographic column chart of death outcome in oxaliplatin-associated neurological AEs.

SUPPLEMENTARY FIGURE S3 The hierarchical structure of preferred term (PT), high level term (HLT), high level group term (HLGT), and system organ class (SOC).

## Abbreviations

AEs, adverse events; FAERS, the Food and Drug Adverse Event Reporting System; TTO, Time-to-onset; PT, preferred term; SOC, system organ class; HLGT, high level group term, HLT, high level term; PS, primary suspect; OR, odds ratio; ROR, reporting odds ratio; PRR, proportional reporting ratio; IC, information component; EBGM, empirical Bayes geometric mean; WSP, Weibull shape parameter; IQR, interquartile range; PPIs, proton pump inhibitors; NSAIDs, nonsteroidal anti-inflammatory drugs.
