# Supplementary figures and images for: Neurological adverse events associated with oxaliplatin: A pharmacovigilance analysis based on FDA adverse event reporting system
Source: Front Pharmacol. 2024 Jul 9;15:1431579. doi: 10.3389/fphar.2024.1431579 (PMC11263116; doi:10.3389/fphar.2024.1431579)

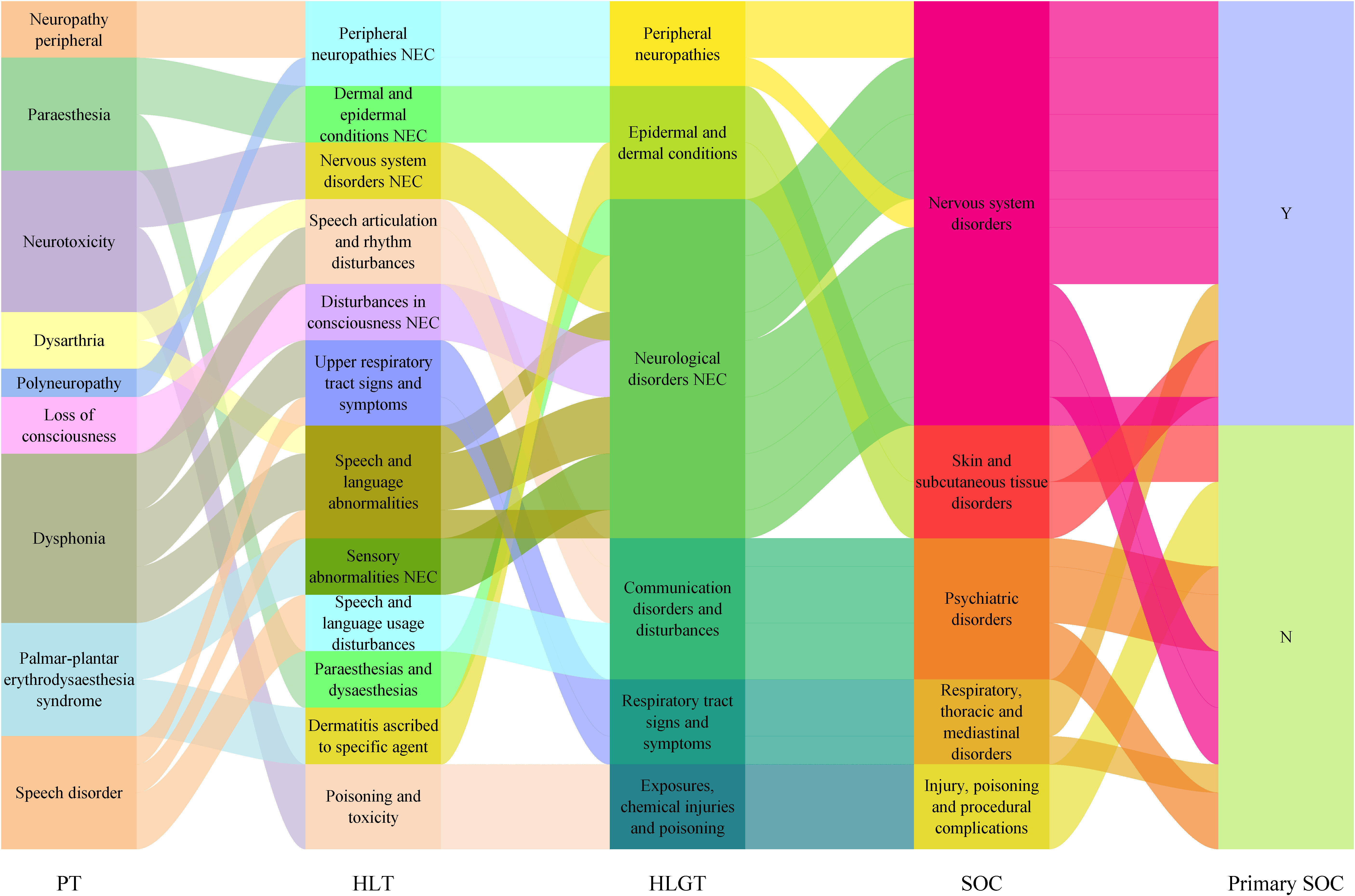

Supplement: Supplementary file 2 [file Image3.JPEG]

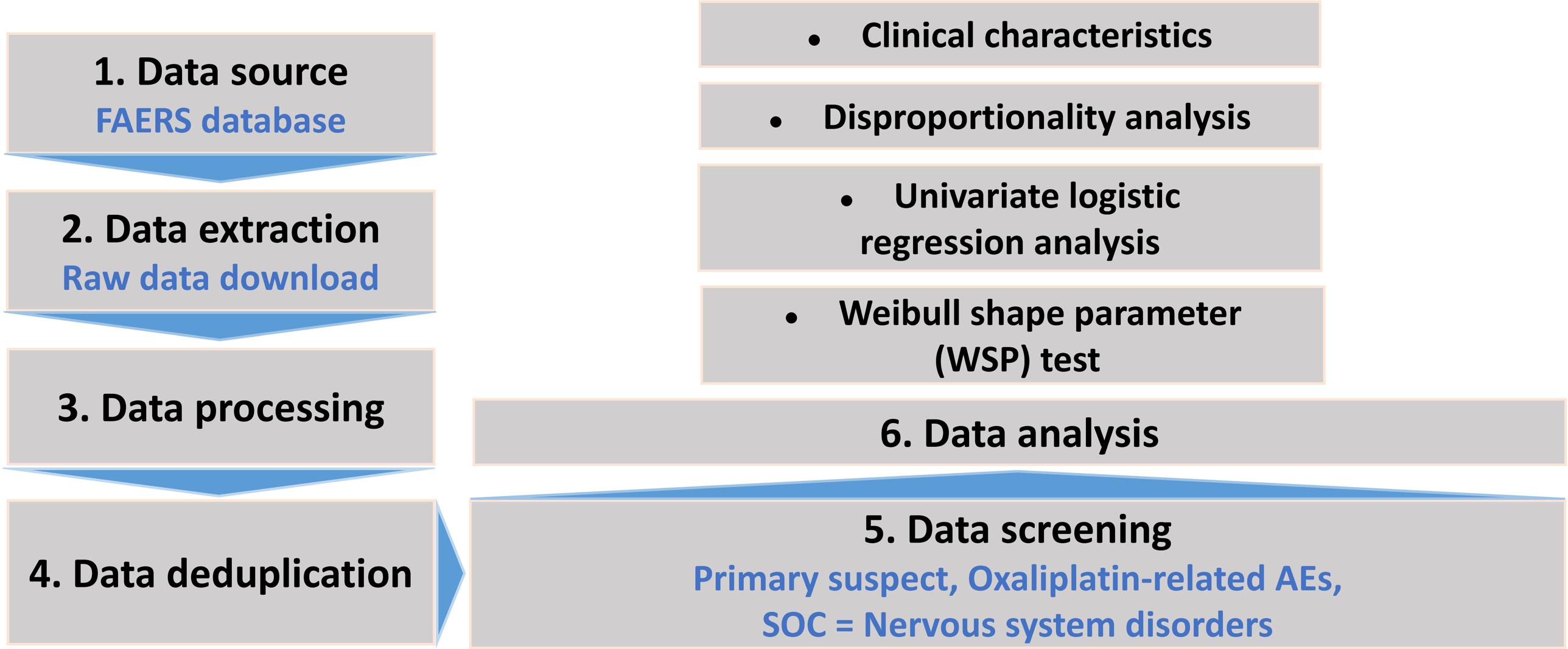

Supplement: Supplementary file 4 [file Image1.JPEG]

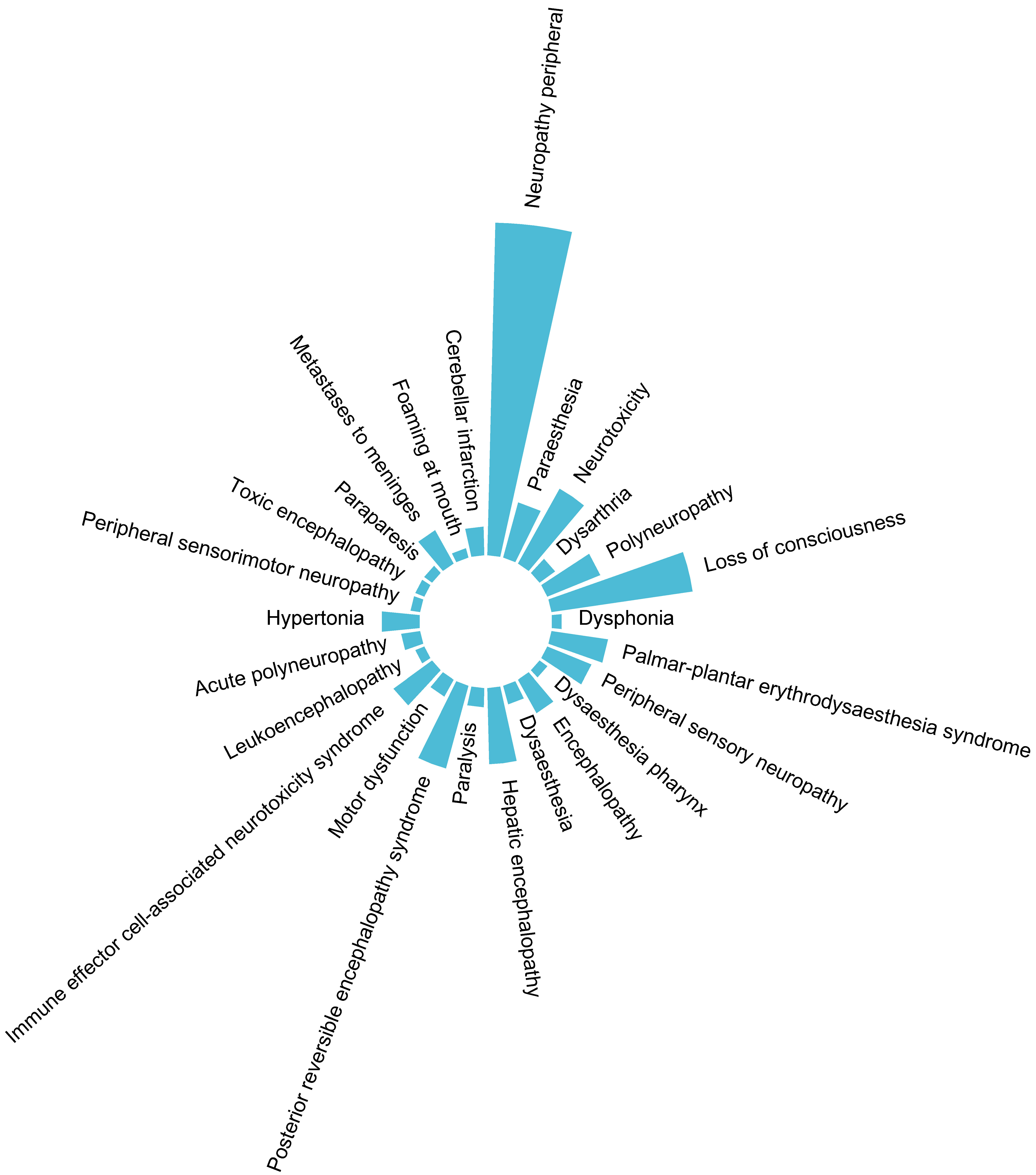

Supplement: Supplementary file 5 [file Image2.JPEG]
